# CARVEDILOL AS PRIMARY PROPHYLAXIS FOR GASTRIC VARICEAL BLEEDING IN PORTAL HYPERTENSION MODEL IN RATS

**DOI:** 10.1590/0102-672020200003e1525

**Published:** 2020-12-16

**Authors:** Andressa de Souza BERTOLDI, Camila Roginski GUETTER, Gabriel Antonio COLTRO, Larissa Maria VOSGERAU, Laura Maria Viscardi BRIGHENTI, Natália Izycki FAUAT, Fernando Bermudez KUBRUSLY, Camila Aparecida Moraes MARQUES, Luiz Fernando KUBRUSLY

**Affiliations:** 1 Mackenzie Evangelical Faculty of Paraná - FEMPAR, Curitiba, PR, Brazil; 2 Institute Denton Cooley Brazil - IDC, Curitiba, PR, Brazil; 3 Federal University of Paraná - UFPR, Curitiba, PR, Brazil; 4 Pontifical Catholic University of Paraná - PUCPR, Curitiba, PR, Brazil; 5 Autonomous University Center of Brazil, Curitiba, PR, Brazil

**Keywords:** Portal hypertension, Beta-adrenergic antagonists, Gastropathy, Hipertensão portal, Antagonistas adrenérgicos beta, Gastropatia

## Abstract

**Background:**

Portal hypertension (PH) can be measured indirectly through a hepatic vein pressure gradient greater than 5 mmHg. Cirrhosis is the leading cause for PH and can present as complications ascites, hepatic dysfunction, renal dysfunction, and esophagogastric varices, characterizing gastropathy.

**Aim:**

To evaluate the use of carvedilol as primary prophylaxis in the development of collateral circulation in rats submitted to the partial portal vein ligament (PPVL) model.

**Method:**

This is a combined qualitative and quantitative experimental study in which 32 Wistar rats were divided into four groups (8 animals in each): group I - cirrhosis + carvedilol (PPVL + C); group II - cirrhosis + vehicle (PPVL); group III - control + carvedilol (SO-sham-operated + C); group IV - control + vehicle (SO-sham-operated). After seven days of the surgical procedure (PPVL or sham), carvedilol (10 mg/kg) or vehicle (1 mL normal saline) were administered to the respective groups daily for seven days.

**Results:**

The histological analysis showed no hepatic alteration in any group and a decrease in edema and vasodilatation in the PPVL + C group. The laboratory evaluation of liver function did not show a statistically significant change between the groups.

**Conclusion:**

Carvedilol was shown to have a positive effect on gastric varices without significant adverse effects.

## Graphical Abstract

[Fig f1a]

**Figure f1a:**
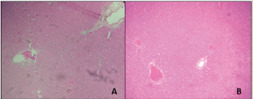
Corte histológico de fígado sem alteração: A) grupo sham-operated + carvedilol; B) grupo LPVP + carvedilol

## RESUMO

**Racional:**

A hipertensão portal (HP), medida indiretamente através do gradiente pressórico da veia hepática >5 mmHg, tem como principal causa etiológica a cirrose. Possui como complicações a ascite, disfunção hepática, disfunção renal e varizes esofagogástricas, que caracterizam o quadro de gastropatia.

**Objetivo:**

Avaliar o uso do carvedilol como profilaxia primária no desenvolvimento da circulação colateral em ratos submetidos ao modelo de ligadura parcial de veia porta (LPVP).

**Método:**

Estudo experimental qualitativo e quantitativo no qual foram utilizados 32 ratos Wistar, divididos em quatro grupos (n=8): grupo I - cirrose + carvedilol (LPVP+C); grupo II - cirrose + veículo (LPVP); grupo III - controle + carvedilol (SO - sham-operated+C); grupo IV - controle + veículo (SO - sham-operated). Após transcorridos sete dias do procedimento cirúrgico, foi administrado carvedilol (10 mg/kg) e veículo (1mL) para os respectivos grupos por sete dias consecutivos.

**Resultados:**

A análise histológica não mostrou alteração hepática em nenhum grupo e diminuição de edema e vasodilatação no grupo LPVP+C. A avaliação laboratorial da função hepática não mostrou alteração com significância estatística entre os grupos.

**Conclusão:**

Carvedilol mostrou ser fármaco com efeito positivo no sangramento das varizes gástricas e sem efeitos adversos significantes.

## Graphical Abstract

[Fig f1as]

**Figure f1as:**
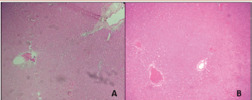
Corte histológico de fígado sem alteração: A) grupo sham-operated + carvedilol; B) grupo LPVP + carvedilol

## INTRODUCTION

Portal hypertension (PH) can be determined indirectly by a pressure gradient of the hepatic vein greater than 5 mmHg. It can occur to cirrhosis or non-cirrhotic causes^11^, the former being the most prevalent. Non-cirrhotic PH generally results from a vascular condition, which can affect both portal and hepatic systems, such as thrombotic portal vein occlusion. On the other hand, cirrhotic PH is associated with an increase in hepatic vascular resistance to blood flow^5^ ^,^ ^9^. PH is the major cause of morbidity and mortality in patients with cirrhosis due to the development of complications, mainly esophagogastric varices^16^, ascites, renal dysfunction, and hepatic encephalopathy^7^. The development of these complications occurs when portal pressure, directly measured, is greater than 10 mmHg and for bleeding when the gradient is greater than 12 mmHg^5^.

In the last 30 years, mortality due to variceal bleeding has decreased significantly. This occurred due to numerous factors, but mainly as consequence of the improvement in the therapeutic options^5^ ^,^ ^6^. The use of non-selective beta-blockers, particularly propranolol, and endoscopic varicose vein ligation are the first-line treatment to prevent bleeding^2^. Because they reduce the hepatic vein pressure gradient, non-selective beta-blockers can be used in both primary and secondary bleeding prophylaxis, in combination with varicose vein ligation or as an alternative to it^8^. However, not all patients present a significant reduction of the hepatic vein pressure gradient with the use of propranolol. Thus, carvedilol has emerged as a promising novel therapy in the treatment of PH^2^.

Carvedilol is a non-selective beta-blocker with vasodilatory properties in addition to anti-alpha1 adrenergic activity and an improvement in the release of nitric oxide^7^. It can also be used as an antioxidant, given its property of inhibiting the production of reactive oxygen species^15^. When compared to propranolol it has shown greater effectiveness as well as with lower rate of non-responder patients^7^.

This study aims to evaluate the use of carvedilol as primary prophylaxis of gastric variceal bleeding in rats submitted to stenosis of the portal vein in the experimental model of partial portal vein ligation (PPVL), as well as dose dependent hepatic toxicity of the drug used.

## METHODS

This is a combined qualitative and quantitative experimental study in which the development of PH was induced by partial portal vein ligation (PPVL). Control groups in the study underwent simulation of operations (sham). The procedures were performed according to the recommendations of the Ethics Committee on Animal Use (CEUA) of the Faculdade Evangélica Mackenzie do Paraná (FEMPAR).

Thirty-two male Wistar rats were used, weighing between 200-300 g. The model chosen for portal hypertension occurred through partial portal vein ligation (PPVL), as described by Sikuler^13^. The animals were divided into four groups: 1) group I: cirrhosis+carvedilol (PPVL+C), submitted to PPVL surgery followed by treatment with carvedilol for seven days starting at day seven after the procedure; 2) group II: cirrhosis+vehicle (PPVL), submitted to PPVL followed by vehicle administration (1 ml normal saline) for seven days starting at day seven after the procedure; 3) group III: control+carvedilol (SO+C), submitted to sham operation without PPVL followed by treatment with carvedilol starting at day seven after the sham procedure; 4) group IV: control+vehicle (SO), submitted to sham operation without PPVL followed by vehicle administration (1 ml normal saline) for seven days starting at day seven after the procedure.

An intramuscular mixture of xylazine hydrochloride 10 mg/kg and ketamine hydrochloride 90 mg/kg was used based on body weight to anesthetize the animals. PPVL was performed through a median laparotomy with posterior exposure of the intestinal loops and of the portal vein. A 20G needle was placed over the portal vein and both joined by a Vicryl^®^ 3.0 thread for partial stenosis of the vein. After withdrawal of the needle and verification that thrombosis had not occurred, the intestinal loops were replaced in the abdominal cavity and moistened with physiological solution, followed by suturing of the cavity by planes. The animals in the control groups (sham operations) underwent the same procedure, with opening of the abdominal cavity and exposure of organs, however without partial ligation of the portal vein.

Treatment with carvedilol started on day 7 after the surgical procedure, and was carried out daily for seven days. It was administered intragastrically at the dose of 10 mg/kg of carvedilol per kg of animal weight. Control animals received the vehicle (normal saline - 0.9% NaCl) in the volume of 1 ml during the same days following procedure.

After 14 days of model development, the animals were again weighed and anesthetized with the same anesthetic mixture previously mentioned. A blood sample was collected through cardiac puncture to perform liver integrity analysis using aspartate-aminotransferase (AST), alanine-aminotransferase (ALT), gamma glutamy-transferase (GGT) and alkaline phosphatase (AF). Afterwards, euthanasia was performed by exsanguination with removal of the stomach, spleen and liver. The organs were weighed (on a precision scale), measured (diameter and volume), and stored. Fragments of the liver and stomach were removed and immersed in a 10% formaldehyde solution for further histological analysis through H&E staining.

### Statistical analysis

The data obtained were included in a spreadsheet (Excel), from which statistical analysis was performed using the statistical software Stata^®^. In descriptive analysis, the data were expressed in measures of central tendency (mean and standard deviation). Inferential statistical analysis was then performed based on the identification of the nature of the variables and number of study groups. The Kruskall-Wallis test was used to compare weights, measures and laboratory tests between the groups. The significance level considered was 5% (95% confidence interval).

## RESULTS

The animals were divided into four groups. Two rats from group IV (SO) died after a few days following the experiment (one due to lesions caused by a physical combat with the other three rats it was kept in the box with, and another due to an unknown cause).

For this reason, these two rats were excluded from the analysis of this study, leaving groups I to III with eight animals each, and group IV with six. When comparing the initial animals weight (Table 1) there was no baseline difference between the groups.

**TABLE 1:** Comparison between the weights of the animals at the beginning of the experiment (mean and standard deviation)

	Group I (n=8)	Group II (n=8)	Group III (n=8)	Group IV (n=6)	p
Initial weight (g)	350.13 (15.62)	350.75 (21.52)	338.63 (21.52)	348.67 (31.80)	0.736

Animals underwent euthanasia by exsanguination on the 7^th^ postoperative day according to the study protocol already described in the methodology section of this study. Post-mortem weights and measures by study group are described in Table 2. No statistically significant difference was seen amongst these variables.

**TABLE 2:** Post-mortem weights and measures (mean and standard deviation)

	Group I	Group II	Group III	Group IV	p
Total weight (g)	374.25 (16.54)	386 (31.95)	366 (31.69)	388 (50.91)	0.7399
Spleen weight (g)	1.34 (0.42)	1.6922 (0.50)	1.67 (0.15)	1.67 (0.26)	0.0759
Spleen length (mm)	25.13 (3.14)	27.6 (2.88)	28.14 (3.13)	30 (2.83)	0.2015
Liver weight (g)	16.71 (2.60)	14.18 (1.17)	13.87 (1.88)	16.92 (1.93)	0.0863
Stomach weight (g)	2.04 (0.62)	2.13 (0.16)	2.33 (0.49)	2.32 (94.75)	0.7153

Regarding the analysis of laboratory tests (Table 3), there was no significant difference between the groups after the intervention period for the values of transaminases, gamma glutamyl-transferase, and alkaline phosphatase.

**TABLE 3:** Laboratory tests for hepatic function after euthanasia (mean and standard deviation)

	Group I	Group II	Group III	Group IV	p
AST (UI/L)	818.63 (285.93)	356.04 (183.14)	652.24 (390.24)	904.10 (222.74)	0.0701
ALT (UI/L)	235.67 (53.12)	201.08 (197.42)	217.57 (144.58)	310.70 (90.80)	0.7656
GGT (UI/L)	0.25 (0.05)	0.18 (0.08)	0.20 (0.11)	0.20 (0.14)	0.5354
AF (UI/L)	28.42 (17.14)	41.64 (31.44)	64.11 (84.30)	72.45 (26.66)	0.2597

**FIGURE 1:**
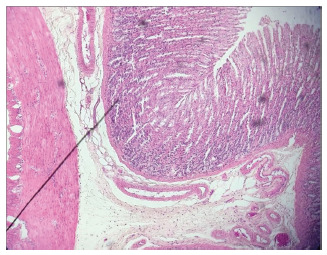
Histological section of the LPVP group showing vasodilation and mucosal edema

**FIGURE 2:**
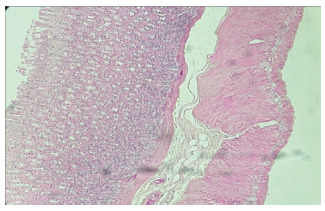
Histological cut of the LPVP+carvedilol group showing attenuation of mucosal edema

In the histological analysis of the gastric mucosa, the development of edema and vasodilatation in the PPVL group (Figure 1) was confirmed by H&E staining, due to the installed angiogenesis process when compared to the SO animals. The LPVP+carvedilol animals (Figure 2) presented attenuation of this phenomenon.

Regarding the analysis of hepatic histology, no alteration of the hepatic tissue was observed in both groups in which carvedilol was used.

**FIGURE 3:**
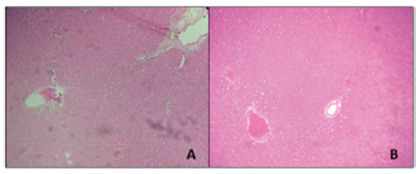
Liver histology showed no change: A) group III (SO+carvedilol); B) group I (PPVL carvedilol)

## DISCUSSION

The partial portal vein ligation model is the most commonly used to study prehepatic PH. Several experimental studies have shown that partial portal vein ligation produces abnormalities equivalent to PH in humans^17^.

In portal hypertension it is observed an increase in vascular resistance in the portal vein and its tributaries. An important drainage route of the portal system to the systemic circulation occurs with the inversion of blood flow in the left gastric vein and the vessels of the submucosa of the stomach and esophagus^4^. Seeking to decrease the pressure, dilation and tortuosity of the veins occurs, leading to a hyperdynamic circulatory state associated with the release of vasoactive substances. In addition, the number of veins in the collateral circulation increases due to angiogenesis^10^.

PH is most commonly caused by liver cirrhosis, followed by schistosomiasis and pre-hepatic portal vein occlusion. Complications from PH include ascites, varicose veins, spontaneous bacterial peritonitis, and variceal bleeding and/or rebleeding^1^. Several studies have shown that the control of PH by pharmacological therapy decreases the rates of complications, especially bleeding, making them preventable^14^.

In the present study, PPVL animals, when submitted to carvedilol treatment, presented reduction of vasodilation and gastric mucosa edema. This finding can be attributed to the potential of carvedilol in reducing the portal pressure. This occurs both through vasodilatory effects and the anti-alpha-1 adrenergic activity and the release of nitric oxide^7^.

When compared to propranolol, carvedilol has shown a greater decrease in the pressure gradient of the hepatic vein, with a reduction greater than 20% of the pressure gradient or to a gradient less than 12 mmHg, thus reducing bleeding rates and complications. In addition, clinical studies have shown that carvedilol has a higher rate of patients responding to the drug, both in the acute phase and in the long-term follow-up of six months, making it a good drug option for primary and secondary prophylaxis^5^.

Regarding the results, the analysis of the AST, ALT, GGT and AF enzymes showed no altered behavior in the model used in the study. AST and ALT are directly related to cell injury and necrosis, and AF to hepatobiliary disease. With these results, it is confirmed that there is no alteration of hepatic integrity, characteristic features of the PPVL model, and no hepatic damage. It is also demonstrated that the dose used of the drug did not prove to be hepatotoxic. This finding could not be compared to previous literature due to the absence of studies following the same experimental protocol.

Recent studies have reported a constant evolution in therapeutic options for PH. Currently new medications are being studied as options to reduce PH, among which various classes of antihypertensive drugs, nitric oxide, statins, anticoagulants and other vasoactive substances have been showing promising results^12^.

Greater knowledge and understanding of the pathophysiology of PH have supported and facilitated the development of new therapeutic options. As a result, animal experiments such as the one described in the present study are essential to the continuous development of knowledge and improvement of therapeutics for patients with PH^3^.

## CONCLUSION

Carvedilol in rats undergoing a partial portal vein ligation model for portal hypertension showed attenuated development of edema and vasodilatation in the gastric mucosa as compared to placebo. No differences were seen in laboratory tests for hepatobiliary injury. No adverse effects were seen. 
